# Comparison of the Performance of Two Commercial Genome-Wide Association Study Genotyping Platforms in Han Chinese Samples

**DOI:** 10.1534/g3.112.004069

**Published:** 2013-01-01

**Authors:** Lei Jiang, Dana Willner, Patrick Danoy, Huji Xu, Matthew A. Brown

**Affiliations:** * Department of Rheumatology, Shanghai Changzheng Hospital, The Second Military Medical University, 200003 Shanghai, China; † The University of Queensland Diamantina Institute, University of Queensland, Princess Alexandra Hospital, Brisbane, Australia 4102, and; ‡ Australian Centre for Ecogenomics, School of Chemistry and Molecular Biosciences, University of Queensland, Brisbane, Australia 4072

**Keywords:** Affymetrix 6.0, Illumina OmniExpress, Immunochip, genetic polymorphisms

## Abstract

Most genome-wide association studies to date have been performed in populations of European descent, but there is increasing interest in expanding these studies to other populations. The performance of genotyping chips in Asian populations is not well established. Therefore, we sought to test the performance of widely used fixed-marker, genome-wide association studies chips in the Han Chinese population. Non-HapMap Chinese samples (n = 396) were genotyped using the Illumina OmniExpress and Affymetrix 6.0 platforms, whereas a subset also were genotyped using the Immunochip. Genotyped markers from the Affymetrix 6.0 and Illumina OmniExpress were used for full genome imputation based on the HapMap 2 JPT+CHB (Japanese from Tokyo, Japan and Chinese from Beijing, China) reference panel. The concordance between markers genotypes for the three platforms was very high whether directly genotyped or genotyped and imputed single nucleotide polymorphisms (SNPs; >99.8% for directly genotyped and >99.5% for genotyped and imputed SNPs, respectively) were compared. The OmniExpress chip data enabled more SNPs to be imputed, particularly SNPs with minor allele frequency >5%. The OmniExpress chip achieved better coverage of HapMap SNPs than the Affymetrix 6.0 chip (73.6% *vs.* 65.9%, respectively, for minor allele frequency >5%). The Affymetrix 6.0 and Illumina OmniExpress chip have similar genotyping accuracy and provide similar accuracy of imputed SNPs. The OmniExpress chip however provides better coverage of Asian HapMap SNPs, although its coverage of HapMap SNPs is moderate.

Genetic polymorphism is known to contribute to phenotypic variation, disease risks, and an individual’s response to pharmaceuticals and the environment. For the past 20 years, genetic linkage combined with positional cloning has achieved tremendous success for mapping the variations that underlie monogenic Mendelian diseases (Jimenez-Sanchez *et al.* 2001). However, it was only with the development of the genome-wide association study (GWAS) approach that significant progress was made mapping disease-associated loci in common complex human diseases (Wang *et al.* 2007). Since the advent of the GWAS era with the publication of the Wellcome Trust Case Control Consortium studies in 2007 (The Wellcome Trust Case Control Consortium 2007), GWAS has become a routinely used tool to identify common, low-risk variants associated with or causative of a wide variety of human diseases. This has led to the robust identification of more than 500 loci associated with common human diseases, representing a huge leap in our understanding of the aetiopathogenesis of human disease (Hindorff *et al.* 2009; Manolio *et al.* 2009).

The GWAS approach has matured considerably since its introduction. The key technical advances have come about through refinement of single-nucleotide polymorphism (SNP) selection on chips, marked increases in SNP density, the development of copy number variant tagging approaches, and improvements in chip throughput and reduction in per SNP genotyping costs. At the moment, Illumina (http://www.illumina.com) and Affymetrix (http://www.affymetrix.com) microarrays are the two most popular microarray platforms in the GWAS research area. The ability to identify associations depends heavily on the coverage of the genotyping chip used (Barrett and Cardon 2006). This coverage varies significantly between major ethnic groups and can be assessed *in silico* using previously genotyped samples of known ethnicity, typically from the HapMap study (Barrett and Cardon 2006; The International Hapmap Consortium 2005; Pei *et al.* 2008). This approach does not take into account real-world factors, notably the genotyping success rate of the different platforms and chips.

To date, most GWAS studies have been performed in populations of European descent. Interest in gene-mapping in Asian and African populations in particular has increased recently, driven at least partly by the fact that the so-called “low-hanging fruit” already have been identified for most common human diseases and the increasing evidence of specificity of a significant number of genetic associations to individual ethnic groups (*e.g.*, Cai *et al.* 2011; Carty *et al.* 2011; Noguchi *et al*. 2011; Smith *et al.* 2011; Yoon *et al.* 2012). This is particularly the case in East Asia, where large case collections have already been established, or where high-quality health services in large populations enable the establishment of suitable case collections for GWAS studies. Transethnic mapping, or comparing association findings in ethnically remote populations, can be highly informative about established loci in addition to identifying novel loci (Hughes *et al.* 2011). When disease associations are shared (with the same polymorphisms) between ethnically remote groups, this implies that the same causal variant is in linkage disequilibrium with the genotyped variant in each population, indicating a common founder mutation (Franceschini *et al.* 2012; Twee-Hee Ong *et al.* 2012).

Here, we test the coverage of two commonly used genotyping chips, the Affymetrix SNP Array 6.0 (abbreviated as Affymetrix 6.0) and Illumina Human OmniExpress Bead Chip (abbreviated as OmniExpress), in a Han Chinese population (n = 396). The Affymetrix 6.0 is designed to genotype more than 906,600 SNPs and 946,000 copy number variants. The OmniExpress is an Illumina Infinium HD BeadChip, which is designed to genotype 733,202 markers per sample. A subset of individuals also was genotyped using the Immunochip, an Illumina Infinium chip containing 196,524 polymorphisms for known immunogenetic loci (Cortes and Brown 2011). Chip performance was assessed in relation to data quality and genomic coverage, both for directly genotyped and imputed SNPs. Genotype concordance was evaluated between the Affymetrix 6.0 and OmniExpress, as well as between these two platforms and a third genotyping platform, the Immunochip.

## Materials and Methods

### Study samples

This study was approved by the research ethics committee of the Second Military Medical University, China. Blood samples were obtained from patients attending outpatient clinics in Changzheng Hospital, Shanghai, China. All the study subjects provided signed informed consent. Subjects genotyped with the Affymetrix 6.0 platform were a subset of a larger cohort of non-HapMap Han Chinese individuals at Changzheng Hospital, which included rheumatoid arthritis cases (n = 51) and controls (n = 188). Only control subjects were genotyped further using OmniExpress or Immunochip. Genomic DNA was extracted from peripheral blood leukocytes using the AxyPrep Blood Genomic DNA Miniprep Kit (Axygen, Union City, CA) according to the manufacturer’s instructions. All genomic DNA was resuspended in TE Buffer for the following study.

### Genotyping

The concentration of genomic DNA was measured using the Quant-iT PicoGreen dsDNA assay (Invitrogen, Carlsbad, CA), and concentrations were standardized to 50 ng/μL for genotyping. All samples were genotyped initially using the Illumina OmniExpress (Illumina Inc., San Diego, CA) and Affymetrix 6.0 (Affymetrix Inc., Santa Clara, CA) chips. Correlation of genotypes was then checked with a further Illumina Infinium chip, the Immunochip (Illumina Inc.), which contains a mixture of common and rare variants (Cortes and Brown 2011). All platforms were operated according to the manufacturer’s instructions.

### Genotype clustering and quality control

Illumina genotype clustering was performed using Illumina’s BeadStudio software (Illumina Inc.). SNPs were reclustered using the study samples, low quality subjects were removed, and a subset of clustering results were manually inspected and verified. All SNPs with GenCall scores less than 0.15 were excluded. For Affymetrix chips, clustering was performed using the program Birdseed version 2, as implemented using Affymetrix Genotyper Console v4.0 (Affymetrix Inc.), using the default quality control (QC) thresholds. Population stratification was assessed with Eigenstrat and SmartPCA (both available from http://genepath.med.harvard.edu/∼reich/Software.htm) using the HapMap phase 3 Release 27 data (Price *et al.* 2006). This identified three ethnic outliers, which were removed before further analysis. Genotyped SNPs were evaluated using PLINK version 1.07 (pngu.mgh.harvard.edu/∼purcell/plink/), and markers with call rates less than 95%, minor allele frequency (MAF) less than 1%, and Hardy-Weinberg Equilibrium *P*-values less than 10^−6^ as were excluded from further analysis (Purcell *et al.* 2007). Genotyping data in PLINK format has been deposited at the European Genome Phenome Archive (EGA, Genome-Phenome http://www.ebi.ac.uk/ega/) which is hosted at the European Bionformatics Institute, under accession number EGAS00000000131.

### Imputation

Imputations were carried out using MACH (www.sph.umich.edu/csg/abecasis/MACH) with 50 rounds of model building (Li *et al.* 2010). The phase 2 JPT+CHB (Japanese from Tokyo, Japan and Chinese from Beijing, China) HapMap Release 27 data were used as the reference panel. Imputation quality was assessed using the R^2^ metric. Imputed SNPs with R^2^ less than 0.3 were considered to be of poor quality and were excluded from analysis of imputation efficacy and genotyping concordance. MACH output files were converted to PLINK format using the free software GenGen [www.openbioinformatics.org/gengen/ (Wang *et al.* 2007)]. Coverage was calculated as the number of genotyped plus imputed SNPs (r^2^ > 0.8) divided by the number of genotyped HapMap markers for the JPT+CHB reference panel at a given MAF. Corrected coverage was calculated using the formula presented in Barrett and Cardon (2006), which adjusts coverage estimates for the total number of polymorphisms in the genome.

### Concordance analysis

**Figure 1:**
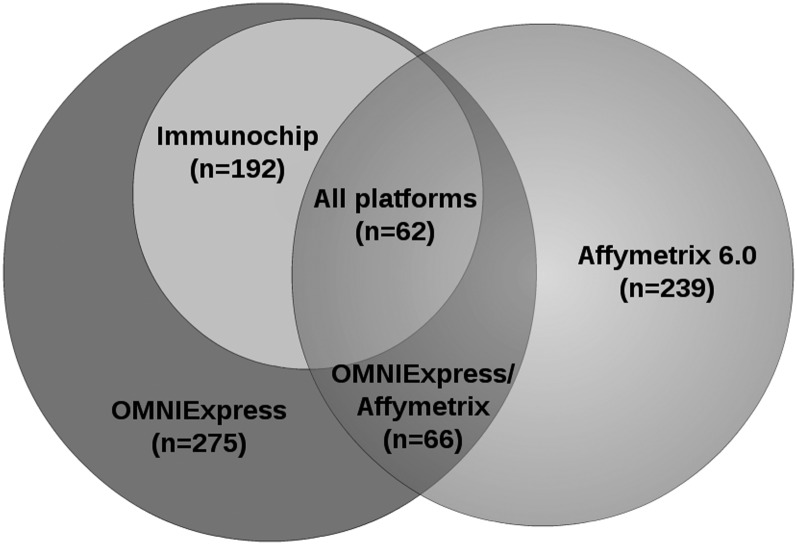
Study sample size. The total number of individuals genotyped by each platform is indicated under the platform name. Sample sizes in overlap regions represent the number of study subjects genotyped by two or more platforms.

Genotype concordance was analyzed using PLINK version 1.07 with the merge function in merge-mode 7, which compares concordance ignoring missing genotypes (Purcell *et al.* 2007). Prior to comparison SNPs genotyped on different platforms were adjusted for strand differences and allele coding to allow for accurate assessment of homozygote to homozygote miscalls as discussed in Results. Figure 1 shows the number of individuals genotyped by each pair of platforms, and Table 1 shows the number of shared markers. Types of discordance, MAF, and R^2^ values for each SNP were extracted from PLINK and GenGen files using Perl scripts. Correlations between concordance, MAF, R^2^ were analyzed using the R function cor.test, and scatterplots were made using the R plot function (R Development Core Team 2005).

**Table 1:** Concordance between genotyped SNPs

Array 1	Array 2	No. Shared Genotyped SNPs Passing QC with MAF > 0.01	Percent Concordance
Affymetrix 6.0	OmniExpress	146,885	99.89
Affymetrix 6.0	Immunochip	13,644	99.89
OmniExpress	Immunochip	18,859	99.97

The third column indicates the number of SNPs shared by Array 1 and Array 2 after quality and MAF filtering. Concordance rates presented in the fourth column represent the sum of counts of concordant markers for each subject divided by the total number of markers for all subjects. MAF, minimum allele frequency; SNP, single-nucleotide polymorphism.

## Results

### Genotyping success and concordance across chips

A total of 396 subjects were genotyped, with subsets subjects genotyped on one, two, or all three platforms (Figure 1). For each array type, data were first subjected to QC to remove SNPs with low call rates and MAF less than 1%. For the Affymetrix 6.0 array, 582,284 SNPs (62.28% of total markers) passed QC and had MAF greater than 1% for each array. The number of SNPs that were successfully genotyped by the OmniExpress chip was 593,582 (81.19% of total markers), which was slightly greater than for Affymetrix 6.0. For the Immunochip, 82,084 markers had MAF less than 0.01, and an additional 2,388 failed QC, leaving 112,112 (57.04% of total markers) genotyped markers. Genotype concordance between chip types was very high for all pairs of platforms; dually genotyped SNPs were more than 99.8% concordant in all cases (Table 1). No significant differences were noted between pairwise concordance, indicating that for genotypes that passed QC, genotyping accuracy did not vary substantially between the different platforms.

### Imputation efficacy

Information on imputation efficacy for HapMap SNPs using genotyped Affymetrix 6.0 and OmniExpress markers is presented in Table S1. The number of imputable SNPs refers to the number of SNPs in the HapMap II CHB+JPT data set for a particular chromosome (Table S1A and B, second column). Imputation efficacy was initially evaluated using the MACH default value of r^2^ =0.3 as in Nothnagel *et al.* (2009). Overall, imputations were more efficacious using the OmniExpress chip, with 4.59% of SNPs failing QC *vs.* 7.10% for the Affymetrix 6.0. For both data sets, there was a high prevalence of SNPs with MAF less than 1%, but this was greater for the Affymetrix 6.0 chip (8.42%) than the OmniExpress (4.72%).

### Coverage of HapMap SNPs

Imputed SNPs also were evaluated at r^2^ = 0.80 to calculate coverage of HapMap CHB+JPT markers (Table 2). The HapMap Phase II CHB+JPT panel contains 2,133,507 markers with MAF greater than 5%. Using the OmniExpress platform, 25.3% of these were genotyped, whereas 52.9% were successfully imputed, giving a naïve coverage rate of 78.2%. Using Barrett and Cardon’s corrected formula for coverage with an estimate of 7.5 million SNPs in the genome, we calculated coverage as 73.0% (Barrett and Cardon 2006), which is much lower than the 91% coverage for markers with MAF greater than 5% reported by Illumina for JPT+CHB populations (www.illumina.com/products/human_omni_express.ilmn). Corresponding coverage estimates for HapMap SNPs with MAF greater than 1% were 76.1% (uncorrected) and 71.1% (corrected; Table 2). Using the Affymetrix platform, we found that the empirical coverages for SNPs with MAF greater than 5% were 66.6% (uncorrected) and 59.2% (corrected), and for MAF greater than 1% were 66.4% (uncorrected) and 57.3% (corrected). This observed coverage was much lower than the theoretical coverage of 84% for JPT+CHB reported in Li *et al.* (2008).

**Table 2:** Coverage of JPT+CHB HapMap 2 SNPs

	No. JPT+CHB HapMap SNPs	HapMap SNPs Genotyped	HapMap SNPs Imputed with r^2^ > 0.8	Coverage	Corrected Coverage
MAF > 0.05					
OmniExpress	2,133,507	540,163 (25.3%)	1,128,626 (52.9%)	78.2%	73.0%
Affymetrix 6.0	2,133,507	501,993 (23.5%)	919,764 (43.1%)	66.6%	59.2%
MAF > 0.01					
OmniExpress	2,344,748	581,788 (24.8%)	1,288,127 (54.9%)	79.7%	71.1%
Affymetrix 6.0	2,344,748	512,669 (21.9%)	1,045,048 (44.6%)	66.5%	57.3%

Only SNPs with a genotyping rate greater than 0.95 were considered. Coverage was calculated as the number of genotyped plus imputed SNPs (r^2^ > 0.8) divided by the number of genotyped HapMap markers for the JPT+CHB reference panel at a given MAF. Corrected coverage was calculated using the formula presented in Barrett and Cardon (2006), which adjusts coverage estimates for the total number of polymorphisms in the genome. MAF, minimum allele frequency; SNP, single-nucleotide polymorphism.

### Concordance of imputed SNPs

The number of imputable SNPs, *i.e.*, the number of SNPs for one platform that can be imputed using a second platform, was calculated as the number of unshared (not dually genotyped) markers with phasing information in the HapMap II CHB+JPT set (Table 3). Imputation efficacy between platforms corresponds to the percentage of imputable SNPs which passed imputation QC (r^2^ > 0.3, MAF > 0.1, call rate > 0.95; Table 4). The OmniExpress platform was more successful at imputing Affymetrix 6.0 markers than vice versa; however, both platforms had similar efficacy for imputation of Immunochip SNPs.

**Table 3:** Imputable SNPs for each array

Array 1	Array 2	No. SNPs Passing QC with MAF > 0.01 Genotyped on Array 1 Only	No. Imputable SNPs Using HapMap CHB+JPT Reference Set (Percent)
Affymetrix 6.0	OmniExpress	435,399	372,433 (85.53%)
OmniExpress	Affymetrix 6.0	446,697	391,758 (87.70%)
Immunochip	Affymetrix 6.0	28,305	25,844 (91.31%)
Immunochip	OmniExpress	23,370	20,135 (86.16%)

To calculate the number of imputable SNPs, SNPs dually genotyped by two arrays were first removed from consideration, as well as any SNPs failing QC. The number of remaining markers with phasing information in the HapMap CHB+JPT set (the reference set for imputations) was then tabulated. The percentages in the fourth column are the number of SNPs with phasing information divided by the number of non-shared SNPs (third column). MAF, minimum allele frequency; QC, quality control; SNP, single-nucleotide polymorphism.

**Table 4:** Concordance and types of discordance in dually genotyped or genotyped and imputed SNPs

Platform 1	Platform 2	Concordance (No. SNPs)	Homozygote to Homozygote	Heterozygote to Homozygote	Homozygote to Heterozygote
Affymetrix 6.0	OmniExpress	99.89% (146,885)	45 (0.2%)	12486 (64.8%)	6750 (35.0%)
Affymetrix 6.0	OmniExpress Imputed	99.55% (252,679)	388 (0.3%)	55336 (44.1%)	69891 (55.6%)
Affymetrix 6.0	Immunochip	99.89% (13,644)	35 (3.9%)	466 (51.5%)	403 (44.6%)
OmniExpress	Affymetrix 6.0 Imputed	99.52% (190,555)	853 (0.3%)	55336 (44.1%)	69891 (55.6%)
OmniExpress	Immunochip	99.97% (18,859)	7 (0.8%)	343 (39.2%)	524 (60.0%)
Immunochip	Affymetrix 6.0 Imputed	99.66% (19,727)	73 (2.2%)	1551 (45.7%)	1769 (52.1%)
Immunochip	OmniExpress Imputed	99.77% (15,024)	186 (1.4%)	6056 (47.6%)	6492 (51.0%)

The number of SNPs exhibiting heterozygote to homozygote mismatch *vs.* homozygote mis-calls are indicated. Percentages indicate the percent of discordant calls which fall into each category. SNP, single-nucleotide polymorphism.

Concordance between imputed and genotyped SNPs for pairs of array platforms was very similar to concordance between pairs of genotyped SNPs (Table 1 and Table 4). Concordance rates exceeded 99.5% in all cases, and no differences were noted in the concordance rates for different pairs of genotyping arrays. This finding suggests that imputation is sufficiently robust to permit combining imputed and genotyped SNPs in studies using both Affymetrix 6.0 and OmniExpress arrays.

Discordance between imputed and genotyped markers was classified into three categories: (1) a heterozygote mis-called as a homozygote, (2) a homozygote mis-called as a heterozygote, and (3) a homozygote called as the opposite homozygote. For all chip types, homozygote mis-calls as heterozygotes were more common than the converse (Table 4). Homozygote-to-homozygote mis-calls were uncommon with all chip types, comprising less than 5% of miscalls in all cases, and occurred slightly less frequently in the imputed OmniExpress data than the imputed Affymetrix 6.0 data (Table 4). Interestingly, homozygote mis-calls were noted between the Illumina OmniExpress and Immunochip arrays, which use the same strand designation system (Table 4).

### Correlation between concordance, MAF, and R^2^

**Figure 2:**
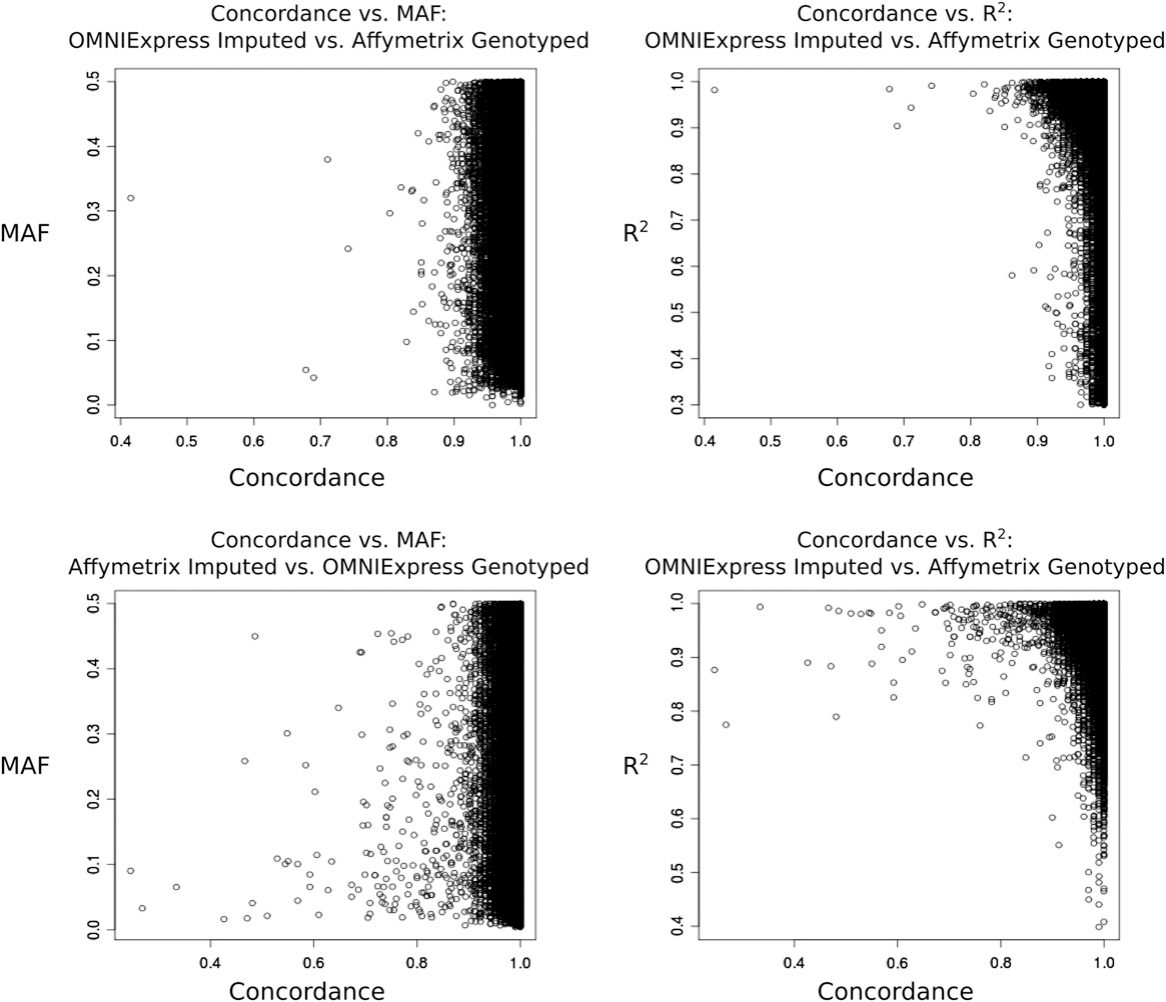
Concordance *vs.* MAF and R^2^ for genotyping platforms. Each data point represents a genetic marker genotyped on one platform and imputed by the other. Concordance represents the proportion of subjects for which the directly obtained and imputed genotypes were the same, and MAF and R^2^ were calculated using PLINK.

Concordance was analyzed on a per-marker basis to explore correlations with MAF and R^2^, as well as to determine the types of discordance present. It has been previously reported that imputation accuracy is positively correlated with MAF, which implies that concordance between genotyped and imputed markers also should increase with increasing MAF (Pei *et al.* 2008; Marchini and Howie 2010). Here, we observed small but statistically significant positive correlations between MAF and concordance. A statistically significant but negligible correlation was observed between concordance and both MAF (Pearson’s r = 0.07, *P* < 0.001) and R^2^ (r = −0.09, *P* < 0.001) were detected when comparing the imputed OmniExpress SNPs to the Affymetrix 6.0 genotyped SNPs (Figure 2, top panels). Similarly, a negligible correlation was observed between concordance and MAF (r = −0.04, *P* < 0.001) for the imputed Affymetrix 6.0 markers compared with OmniExpress genotypes (Figure 2, bottom panels). The correlation between R^2^ and concordance was greater (r = −0.12, *P* < 0.001), but still quite low.

## Discussion

In this study we have compared the real-world performance of two widely used genotyping platforms, Affymetrix 6.0 and Illumina OmniExpress, in a non-HapMap Han Chinese population. Concordance between genotyped markers across the Affymetrix and Illumina platforms, including the Immunochip, was high, with no significant differences noted between different pairs of chips. Nothnagel *et al.* showed similarly high rates of concordance between the Affymetrix 6.0 and Illumina 550k platforms for genotyping in a German population (Nothnagel *et al.* 2009). Concordance of imputed with directly genotyped SNPs was slightly lower than between genotyped SNPs alone, but the level of discordance was still quite low. Although this discordance is minor, care must be taken when combining information from different genotyping platforms for cases and controls because platform-specific differences can lead to spurious or inflated associations (Sinnott and Kraft 2011). Low MAF had little practical impact on the concordance or imputation quality as assessed by R^2^, suggesting that these GWAS chips, although not specifically designed to capture rare variants, may actually capture a significant fraction of genetic variation due to low frequency variants.

Although discordance between both directly genotyped and imputed and genotyped SNPs was low, we noted differences in the type of discordance, with a greater frequency of homozygote genotypes being imputed as heterozygotes than the converse. A slightly greater error rate was observed when comparing imputed data from Affymetrix 6.0 chips with either the OmniExpress or Immunochip data, than with genotypes imputed from OmniExpress compared with directly genotyped Affymetrix 6.0 or Immunochip SNPs. This finding suggests that the imputation accuracy from Affymetrix SNPs is lower than from OmniExpress, although the difference was small. Imputation accuracy was lower on average for Affymetrix platforms as compared to Illumina platforms for CHB+JPT populations in a previous study by Li *et al.* (2008).

Significant differences were noted in the ability to impute from the OmniExpress and Affymetrix 6.0, and imputation efficacy using MACH was much lower in general than previously reported [16]. Many imputed SNPs were not considered for further analysis due to low genotyping rates, poor QC values, and MAF less than 1% in the target panel (Table 2). Xu *et al.* (2009) demonstrated the presence of significant substructure in Han Chinese populations, with notable differences between Northern and Southern Han subpopulations. The HapMap CHB+JPT reference panel includes genotypes from Northern Han (Beijing) and Japanese individuals, while our study population was from Shanghai, which is in the Southern Han region. Some markers which are polymorphic in the JPT+CHB reference panel with MAF greater than 1% may be less polymorphic or monomorphic in the Han Chinese population studied here, contributing to the reduced imputation efficacy. Greater imputation efficacy might be achieved by the use of mixed reference panels as described in Huang *et al.* (2009).

Using HapMap data, Illumina reports that OmniExpress chips capture around 91% SNPs with MAF > 5% and r^2^ > 0.8 in CEU (Utah residents with European ancestry from the Centre du Etude Polymorphisme Humain collection) populations, with similarly good performance also in CHB+JPT (91% in HapMap). In our study, coverage was much lower for the OmniExpress platform (Table 3). This decreased coverage was largely attributable to imputation efficacy as described previously. Coverage estimates are known to be inflated when coverage is calculated using on the reference panel from which tagSNPs were selected during the chip design process (Barrett and Cardon 2006). This results in overfitting, which in combination with the small sample size of the HapMap data set can lead to exaggerated coverage estimates (Hao *et al.* 2008). Markers for the Illumina OmniExpress platform were selected from HapMap SNPs representative of common variants (MAF > 5%) which may also resulted in overestimation of coverage (www.illumina.com/products/human_omni_express.ilmn). The use of more specialized reference panels with larger sample sizes, and genotyping platforms tailored to East Asian populations could improve coverage in future studies.

Coverage for the Affymetrix 6.0 chip was lower than for the OmniExpress because a greater proportion of HapMap SNPs could be imputed using OmniExpress. Lower coverage of CEU, JPT+CHB, and YRI populations by Affymetrix arrays as compared with Illumina arrays has previously been reported and may be related to differences in SNP selection between the two platforms (Mägi *et al.* 2007; Li *et al.* 2008, 2010). The Affymetrix microarray technology relies on the differential hybridization of genomic DNA to 25-mer probes which match SNP alleles, while the Illumina Infinium technology uses hybridization followed by primer extension (Kennedy *et al.* 2003; Steemers *et al.* 2006; Ragoussis 2009). Although Illumina emphasizes tagging SNPs, SNP selection for the Affymetrix system is limited by technical constraints. For example, SNPs chosen for the Affymetrix 6.0 assay must be located within fragments generated by a restriction digest by the enzymes Nsp I and Sty I (http://www.affymetrix.com), and are then required to ameliorate to universal hybridization conditions for adaptor ligation and subsequent inclusion on the array (Ding and Jin 2009).

This study demonstrates that, in a real-world setting, both the Affymetrix 6.0 and OmniExpress chips achieve good coverage of genetic variation in Japanese and Chinese as defined by HapMap. From our results, we can conclude that despite the different mechanisms in chemistry, the Affymetrix 6.0 and OmniExpress platforms both give good call rates and similar genotype accuracy, in comparison with the Illumina Infinium Immunochip genotypes. Further, the imputation accuracy comparing SNPs imputed on one platform with SNPs genotyped on another platform was high, indicating that, with appropriate quality control, it is valid to combine imputed and genotyped SNPs in studies where samples have been genotyped either on the Affymetrix 6.0 or OmniExpress chips.

## Supplementary Material

Supporting Information
