# Supplementary material for: Comparison of the Performance of Two Commercial Genome-Wide Association Study Genotyping Platforms in Han Chinese Samples
Source: G3 (Bethesda). 2013 Jan 1;3(1):23–9. doi: 10.1534/g3.112.004069 (PMC3538340; doi:10.1534/g3.112.004069)
Supplement: Supporting Information [file supp_3_1_23__index.html]

Supporting Information 

# Comparison of the Performance of Two Commercial Genome-Wide Association Study Genotyping Platforms in Han Chinese Samples

## Supporting Information for Jiang *et al.*, 2013

**Files in this Data Supplement:**

- Table S1 - Overall imputation efficacy for OmniExpress (A) and Affymetrix 6.0 (B) arrays (PDF, 58 KB)
