# Supplementary material for: Comparison of the Performance of Two Commercial Genome-Wide Association Study Genotyping Platforms in Han Chinese Samples
Source: G3 (Bethesda). 2013 Jan 1;3(1):23–9. doi: 10.1534/g3.112.004069 (PMC3538340; doi:10.1534/g3.112.004069)
Supplement: Supporting Information [file supp_3.1.23_TableS1.pdf]

Table S1 Overall imputation efficacy for OmniExpress (A) and Affymetrix 6.0 (B) arrays.

A)

Chromosome	Imputable SNPs[1]	SNPs below quality[2]	SNPs MAF<0.01[3]	SNPs GENO<0.05[3]	Remaining SNPs
1	186465	9385 (5.03%)	8494	27481	142673 (76.51%)
2	206699	7508 (3.63%)	9125	29143	163169 (78.94%)
3	164502	6745 (4.10%)	7444	25058	127144 (77.29%)
4	152652	7105 (4.65%)	6661	25157	115711 (75.80%)
5	159272	6429 (4.04%)	7252	24277	123258 (77.39%)
6	177117	7304 (4.12%)	8375	25074	138454 (78.17%)
7	134520	5837 (4.34%)	5854	21670	102909 (76.50%)
8	141590	5946 (4.20%)	6033	21041	110116 (77.77%)
9	118073	4811 (4.07%)	5211	19440	90154 (76.35%)
10	132263	6692 (5.06%)	5979	18956	102286 (77.34%)
11	124385	5701 (4.58%)	5737	17580	96888 (77.89%)
12	120027	8020 (6.68%)	5791	19404	88787 (73.97%)
13	99177	4656 (4.69%)	4668	13960	77136 (77.78%)
14	80221	3675 (4.58%)	3790	12045	61732 (76.95%)
15	69962	3042 (4.35%)	3357	12137	52342 (74.81%)
16	66561	3435 (5.16%)	3069	13605	47547 (71.43%)
17	53365	2374 (4.45%)	2275	9641	39643 (74.29%)
18	73303	3437 (4.69%)	3689	11378	55778 (76.09%)
19	35046	1856 (5.30%)	1685	6906	25324 (72.26%)
20	59192	3513 (5.93%)	2657	10043	43832 (74.05%)
21	33878	1531 (4.52%)	1182	5792	25793 (76.13%)
22	32713	2107 (6.44%)	1551	6280	23414 (71.58%)
**Total**	**2420983**	**111109 (4.59%)**	**109879**	**376078**	**1854090 (76.58%)**

B)

Chromosome	Imputable SNPs[1]	SNPs below quality[2]	SNPs MAF<0.01[3]	SNPs GENO<0.05[3]	Remaining SNPs
1	186465	14102 (7.56%)	14603	28283	116082 (62.25%)
2	206699	12189 (5.90%)	15312	51275	134860 (65.24%)
3	164502	9894 (6.01%)	12613	42163	105630 (64.21%)
4	152652	11312 (7.41%)	11075	41305	94623 (61.99%)
5	159272	9767 (6.13%)	11906	39981	103055 (64.70%)
6	177117	10222 (5.77%)	13840	43395	115510 (65.22%)
7	134520	9258 (6.88%)	9998	35918	84501 (62.82%)
8	141590	8699 (6.14%)	10068	35400	92023 (64.99%)
9	118073	8070 (6.83%)	9057	32882	72629 (61.51%)
10	132263	8630 (5.77%)	10430	33235	84536 (60.52%)
11	124385	7638 (6.14%)	10023	31274	80044 (64.35%)
12	120027	11417 (9.51%)	9502	31284	72486 (60.39%)

13	99177	6549 (6.60%)	7981	25801	62746 (63.27%)
14	80221	5210 (6.49%)	6608	21320	50011 (62.34%)
15	69962	5130 (7.33%)	5753	20975	41123 (58.78%)
16	66561	5786 (8.69%)	5536	21315	37142 (55.80%)
17	53365	5969 (11.19%)	4803	17373	28804 (53.98%)
18	73303	5484 (7.48%)	6222	19246	45267 (61.75%)
19	35046	5594 (15.96%)	3515	11857	16705 (47.67%)
20	59192	5129 (8.67%)	4980	16034	35681 (60.28%)
21	33878	2353 (6.95%)	2539	10271	20138 (59.44%)
22	32713	3550 (10.85%)	3089	10613	17499 (53.49%)
**Total**	**2420983**	**171952 (7.10%)**	**189453**	**641200**	**1511094 (62.42%)**

[1]Imputable SNPs refers to the number of SNPs on each chromosome with phasing information in the HapMap JPT+CHB reference set.

[2]SNPs below quality indicates SNPs with R^2 values less than 0.3.

[3]Minor allele frequency (MAF) and missingness per marker (GENO) were calculated for all imputable SNPs passing the quality filter. Thus, the same SNP may be included in both totals (i.e. some SNPs had both MAF<0.01 and GENO<0.05).
